# Phenotypic and Genetic Variation of an Interspecific *Centaurium* Hybrid (*Gentianaceae*) and Its Parental Species

**DOI:** 10.3390/plants8070224

**Published:** 2019-07-14

**Authors:** Tijana Banjanac, Sanja Đurović, Mihailo Jelić, Milan Dragićević, Danijela Mišić, Marijana Skorić, Jasmina Nestorović Živković, Branislav Šiler

**Affiliations:** 1 Institute for Biological Research “Siniša Stanković”, University of Belgrade, Bulevar despota Stefana 142, 11060 Belgrade, Serbia; 2 Faculty of Biology, University of Belgrade, Studentski trg 16, 11000 Belgrade, Serbia

**Keywords:** *Centaurium*, interspecific hybridization, allopolyploidy, speciation, morphometry, EST-SSR, *trn*L-F

## Abstract

Interspecific hybridization is one of the major actuators of evolutionary changes in plants. As the result of allopolyploid hybridization, offspring may gain different ploidy levels in comparison to parental species, which can provide them instant reproductive isolation. Two tetraploid sister species, *Centaurium erythraea* and *C. littorale*, readily cross-fertilize, resulting in hybrids of various ploidy. In northern Serbia, two stable populations of a hexaploid taxon *C. pannonicum* have been documented. It has been proposed previously that this taxon emerged after an interspecific hybridization event between two tetraploid sister-species: *C. erythraea* and *C. littorale* subsp. *compressum*. The existing populations of the hybridogenic taxon, as well as neighboring populations of the two parental taxa were here characterized by both morphometrics and molecular markers (EST-SSR and *trn*L-F). Three leaf and two flower characteristics were found to be informative in delimitation of the parental taxa and in their discernment from hybrid individuals, the latter having intermediate values. Eight microsatellite markers were found to have good ability to distinguish studied taxa, placing *C. pannonicum* in closer relationship with *C. erythraea*. Conversely, *trn*L-F plastid marker nominated *C. littorale* subsp. *compressum* to be the donor of the *C. pannonicum* plastid DNA. Reproductive isolation of the hexaploid hybrid individuals from the parental species should be examined as the next logical step in describing the new species.

## 1. Introduction

The significance of interspecific hybridization in the “origin” of plant species and evolution has been debated for decades, with opposing views on it regarding it as a creative evolutionary force or as an evolutionary noise [1]. Since the reproductive barriers between morphologically well-described congeneric species are often weak, interspecific hybridization can occur quite frequently, which has been a focus of many botanists for a very long time [2].

It is a well-known fact that a new hybrid line can be formed through allopolyploidization or homoploid hybrid speciation, the former being more common and more feasible [1]. A recent article [3] suggests that 11% of the 47 therein studied plant species most likely have an allopolyploid origin, while Mallet [4] estimates that about 25% of plant species are known to hybridize with at least one other species.

Huge numbers of plant species have been reported to have passed through at least one whole genome duplication event [5], and immense angiosperm diversity is assumed to originate from recurrent polyploidization events [6]. In between, polyploids may step into interspecific crossing, which may result in occurrence of hybrids harboring multiple sets of parental genomes.

The genus *Centaurium* Hill (family *Gentianaceae*) includes about 25 annual or biannual species having either diploid or polyploid genomes [7]. They are of Mediterranean origin, and were mostly spread to the north and west during the last few glaciations [8]. Different species and subspecies of this genus prefer similar habitats and are often found in sympatry, where they are “pleasing” to hybridize between themselves [9,10,11,12,13]. Interspecific hybridization represents one of the most important causes of phenotypic variations within the genus, causing appearance of hybrid swarms of morphologically intermediate appearance. Such prominent morphological polymorphism has led botanists to define an excessive number of species names within the genus [7,14,15,16,17,18,19], which further triggered nomenclatural problems and divergence in taxonomic approaches [7,20,21,22]. Back in 1948, Anderson [23] noticed that “the gene flow from one species to another may go far beyond any point which could be detected by ordinary morphological techniques”. Being additionally influenced by the environment, the morphological characteristics of the genus *Centaurium* are poorly featured in species determination and delimitation [7,11,16,20,24]. However, combination of morphological characteristics and newly developed molecular techniques may provide a solid basis for overcoming these difficulties. Morphometric methods are highly welcome in modern botanical studies to contribute in covering a wide picture of species variation, thus supporting or rebutting results obtained by cutting-edge molecular techniques. So far, different methods have been used in population genetics, taxonomy, and hybridization studies within the genus *Centaurium*, such as morphometrics and cytogenetics [11,12,13,25,26], or phytochemical profiling [9,27,28,29,30,31,32]. In modern phylogenetics studies, molecular markers are, of course, the most commonly applied [33]. Accordingly, three papers [8,24,34] introduced a comprehensive phylogenetic survey which indicated the polyphyletic origin of the genus *Centaurium* and proposed a new classification, while RAPD [9], ITS and plastid markers (*trn*L-F regions; [10]) have been used to study the interspecific hybridization between several species.

Here we studied the background and the outcomes of the interspecific hybridization between two sister-species: *C. erythraea* Rafn and *C. littorale* (Turner) Gilmour (in this particular case, *C. littorale* subsp. *compressum* (Hayne) Kirschner) (following up-to-date knowledge and achievements of the most relevant taxonomic and nomenclature source, Euro + Med Plantbase [19], the taxon *Centaurium littorale* (Turner) Gilmour contains two subspecies: *Centaurium littorale* (Turner) Gilmour subsp. *littorale* and *Centaurium littorale* subsp. *compressum* (Hayne) Kirschner, with *Centaurium uliginosum* (Waldst. & Kit.) Beck being a synonym for the latter one). They are both found in the continental areas of the central and east Europe [35] and, as we have shown earlier [32], both of them carry tetraploid genomes. Their interspecific hexaploid hybrids, named *C. pannonicum*, grow in stable populations in a few locations in the northern Serbia and were, however, found to be hexaploid [32]. With a hint that this might lead to speciation of this hybridogenic taxon, we aimed at analyzing the genetic basis of both parental taxa and the hybrid populations, implementing newly reported EST-SSR markers for the genus *Centaurium* [36] and analyzing the non-coding regions of the plastid DNA. As a contribution to perceiving the overall phenotypic picture of this interspecific hybridization case, which was started earlier by the phytochemical profiling [32], the divergence between the hybrid and the parental individuals is additionally investigated by a thorough analysis of the key morphological characteristics. 

## 2. Results

### 2.1. Variation of Morphological Parameters

**Figure 1:**
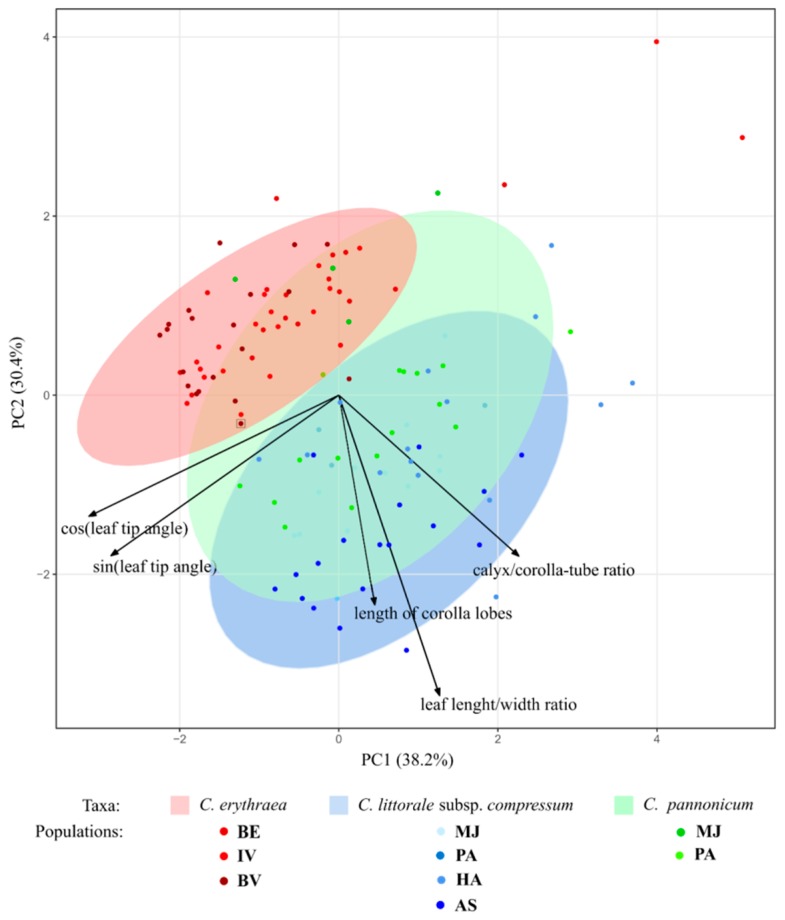
PCA biplot of three *Centaurium* taxa (*C. erythraea*, *C. littorale* subsp. *compressum* and *C. pannonicum*) based on the following characters: length/width ratio of floral stalk leaves, calyx/corolla-tube ratio, length of corolla lobes, and leaf tip angle, which was decomposed to the sine and cosine components. The scatterplot consists of 127 individuals in the plane of the first and the second principal coordinates. Feature loadings were scaled by multiplying with 5 so they could be more readily observed on the graph.

Principal component analysis (PCA) biplot based on five morphological characters of 127 individuals provided good discrimination between two parental species, particularly along the principal component 2 (PC2) (Figure 1). The first two dimensions contribute 68.6% to the total variation and are sufficient based on Horn’s parallel analysis. The cloud comprised of the hybrid individuals is rather scattered, but is positioned between the two clouds belonging to the parental species. Sub-structuring of the tree taxa by the populations is not observed.

**Figure 2:**
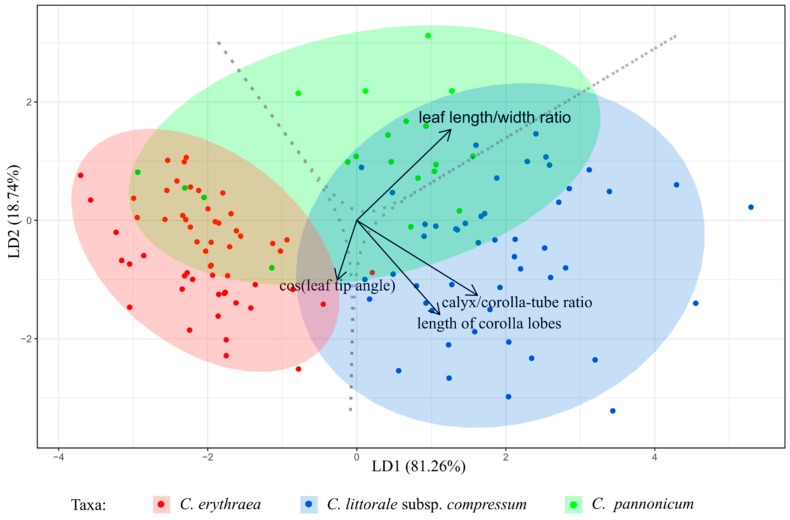
Visualization of LDA of three *Centaurium* taxa (groups were defined as: *C. erythraea*, *C. littorale* subsp. *compressum* and *C. pannonicum*) based on the following characters: length/width ratio of floral stalk leaves, calyx/corolla-tube ratio, length of corolla lobes, and cosine of the leaf tip angle. For graph construction, the LDA model fitted on all the data was used. Model decision bounds are indicated by the dashed lines.

Linear discriminant analysis (LDA) showed that the selected morphological characters were very useful for taxa diversification (Figure 2), particularly between the hybrid individuals and *C. littorale* subsp. *compressum*, which was hardly discoverable by PCA. Based on the sequential backward search, the highest classification accuracy (91.5%) in repeated k-fold cross validation was achieved when LDA used four out of the five provided features (omitting *sin*(leaf tip angle)). The top features in order are: length/width ratio of floral stalk leaves (LDA achieves 65.6% classification accuracy on average on the hold out instances in repeated k-fold cross validation with just this feature) + calyx/corolla-tube ratio (77.8% classification accuracy with the addition of this feature) + length of corolla lobes (89.5% classification accuracy with the addition of this feature) and + *cos*(leaf tip angle) achieving the observed 91.5% accuracy. The highest proportion of falsely classified individuals was from *C. pannonicum* which was predicted as *C. erythraea* on average in 3.2% of total cases and *C. littorale* subsp. *compressum* which was predicted to be *C. pannonicum* on average in 2.7% of total cases (Table S1). 

### 2.2. Genetic Diversity Estimation: Eight-Locus Microsatellite Analysis

Population-genetics parameters are summarized for nine populations in Table 1. The highest number of alleles per population (34) was observed for *C. erythraea* originating in Bački Vinogradi (*C.ery.***BV**) with average 4.25 alleles per locus. The lowest number of alleles was recorded for two populations of *C. littorale* subsp. *compressum*, originating in Majdan and Palić (*C.l.c.***MJ** and *C.l.c.***PA**), both averaging 2.5 alleles per locus. Throughout the populations, 27.33 alleles per population were recorded on average.

Expected heterozygosity ranged from 0.8709 in *C. littorale* subsp. *compressum* (**PA**) to 0.9912 in the *C. pannonicum* (**MJ**) with an average value of 0.9121 throughout the studied populations. Observed heterozygosity was evidently lower for all populations: from 0.5588 in the *C. erythraea* (**BE**) to 0.8125 in the *C. littorale* subsp. *compressum* (**AS**), with an average of 0.6750 (Table 1).

The F_ST_ and Jost’s D indices, which quantify genetic differences between populations, are presented in Table 2. The lowest distances were recorded between three *C. littorale* subsp. *compressum* populations originating in Subotica Sands: Majdan (**MJ**), Palić (**PA**) and Hajdukovo (**HA**), followed by two *C. erythraea* populations from Fruška Gora Mountain (**BE** and **IV**). According to these results, the three populations of *C. littorale* subsp. *compressum* differ at the most from two *C. erythraea* populations from Fruška Gora Mountain and slightly less from the neighboring *C. erythraea* population **BV**. Each of the two *C. pannonicum* populations had considerably lower F_ST_ values *vs*. all the three populations of *C. erythraea* than *vs*. each of *C. littorale* subsp. *compressum*. The same trends can be observed within the Jost’s D values.

**Figure 3:**
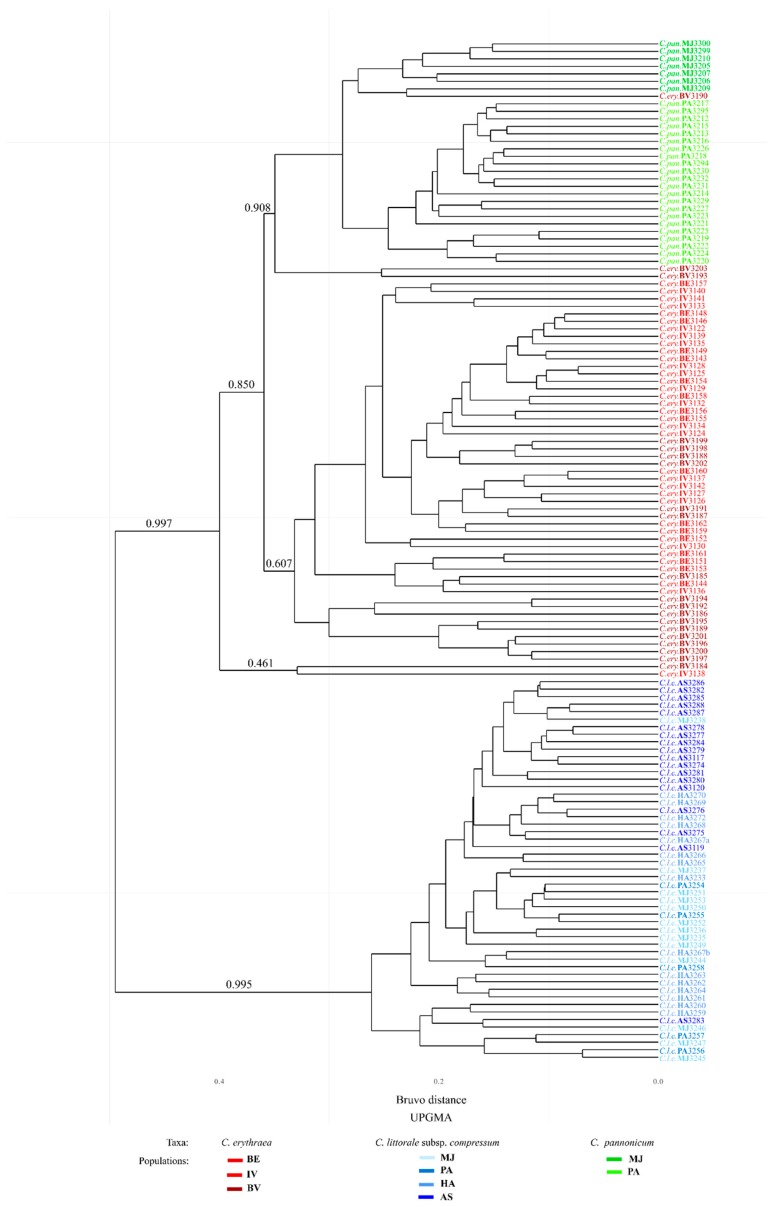
Graphical presentation of the hierarchical cluster analysis (HCA) by UPGMA method based on Bruvo genetic distances, which were obtained from the allele frequencies at eight EST-SSR loci. Bootstrap values are indicated on main clusters and sub-clusters. For population codes, please refer to Table 3.

UPGMA dendrogram constructed from Bruvo genetic distances, which were obtained from the allele frequencies at eight EST-SSR loci (Figure 3), consists of two main clusters which split in Bruvo distance at the value of about 0.5. The first cluster, which is supported by a high (0.995) bootstrap value, is comprised solely of the individuals belonging to *C. littorale* subsp. *compressum*. The cluster is quite coherent, and four populations of this taxon are spread more or less evenly, with no population differentiation visible except that the individuals from the population **AS** tend to cluster together. The second cluster, also having a strong (0.997) bootstrap support, contains two other taxa. A substantial genetic variation of *C. erythraea* is visible in this cluster. Two individuals (*C.ery.***BV** 3184 and *C.ery.***IV** 3138), originating in different populations, are the first to segregate at the Bruvo distance of 0.4. Most of the remaining individuals of the same taxon are further clustering together in a fairly heterogeneous sub-cluster without population diversification. However, the other sub-cluster of the second cluster, comprising mainly hybrid individuals, also contains three “interlopers” from *C. erythraea* (Figure 3). Two of them differentiate earlier from the sub-cluster, with a negligible distance from the *C. erythraea* sub-cluster. The third individual (*C.ery.***BV** 3190) turned out to be misclassified at the beginning of the study (see below). In any case, the mentioned sub-cluster is made of the hybrid *C. pannonicum* individuals with obvious differentiation between its two populations.

**Figure 4:**
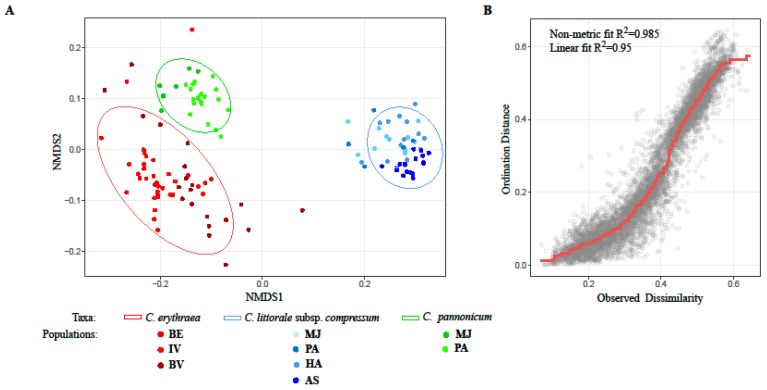
Nonmetric multidimensional scaling (NMDS) based on the Bruvo distances between individuals. (**A**)—NMDS scatterplot; populations are indicated by different colors as shown in the legend. Each taxon is encircled by a 95% confidence ellipse: red—*C. erythraea*, green—*C. pannonicum*, blue—*C. littorale* subsp. *compressum*. (**B**)—Shepard diagram (goodness-of-fit scatterplot) of observed Bruvo distances and NMDS transformed distances. Stress = 0.12.

Non-metric multidimensional scaling (Figure 4) was used to visualize the results obtained from the Bruvo genetic distances among 136 individuals. It showed an evident differentiation of the *C. littorale* subsp. *compressum* group of individuals from the two other taxa, upholding the results obtained by unsupervised UPGMA. Likewise, the second group is comprised of *C. erythraea* and *C. pannonicum* genotypes, the latter one forming its own subgroup.

### 2.3. Variation in trnL-F Plastid Region

The length of the unaligned amplified *trn*L-F region of 43 individuals included in research ranged between 504 and 514 bp. Within this research, it was not possible to read the sequences with primer “c”. The final alignment was done with 43 sequences (Table 3), which were aligned against the 16 *trn*L-F intergenic sequences retrieved from GenBank (Table 4).

**Figure 5:**
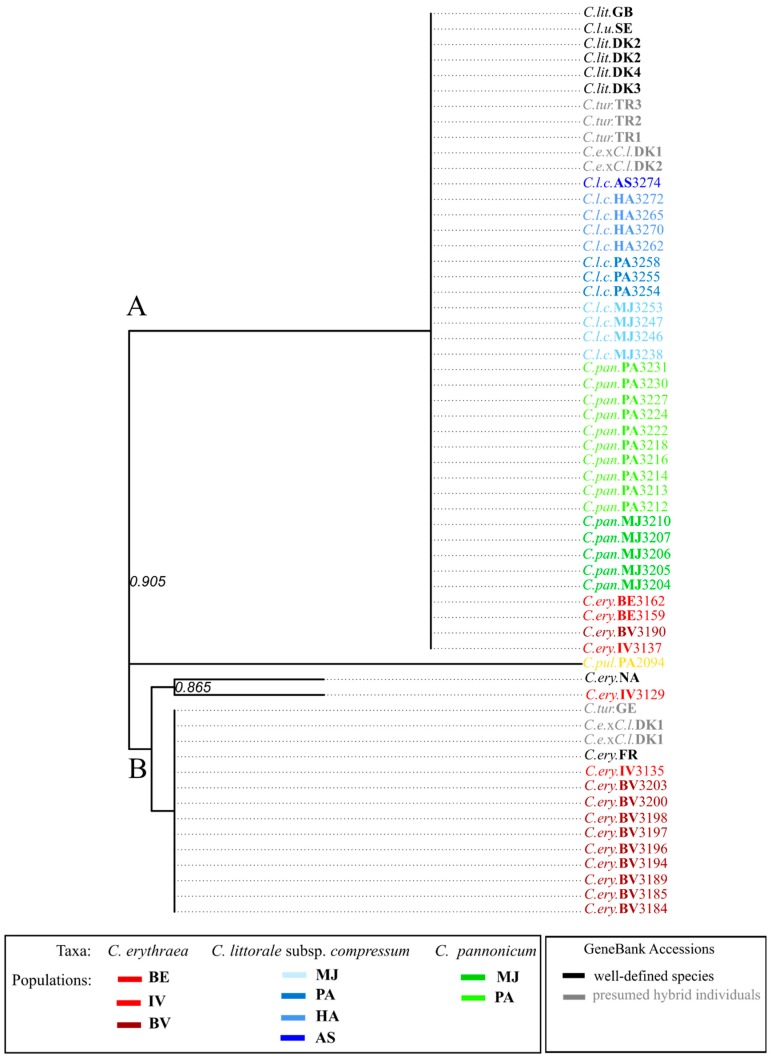
Maximum likelihood phylogenetic tree based on *trn*L-F region of the studied *Centaurium* taxa with *C. pulchellum* as outgroup. Support cluster values, estimated using the Shimodaira-Hasegawa test, are indicated. Sequence tags retrieved from the GenBank are written in black and gray letters while the initials of the country of origin are follows: **GB**-Great Britain, **SE**-Sweden, **TR**-Turkey, **GE**-Georgia, **FR**-France, **DK**-Denmark. For labels please refer to Table 3 and Table 4.

A maximum-likelihood phylogenetic tree of *trn*L-F dataset (Figure 5) revealed two haplotypes in the whole set of sequences. Haplotype A possessed all the individuals characterized as *C. pannonicum*, *C. littorale* subsp. *compressum*, *C. littorale* and *C. uliginosum* (the synonym for *C. littorale* subsp. *compressum*). This haplotype was harbored by 5 more individuals characterized as hexaploid *C. turcicum* and tetraploid *C. erythraea × C. littorale*. Haplotype A also appeared in four individuals of *C. erythraea*, two of which were from **BE** locality, and one from each **IV** and **BV**. 

Haplotype B owned all the remaining sequences belonging to *C. erythraea* individuals as well as another two sequences belonging to the tetraploid hybrid individuals of *C. erythraea × C. littorale* and one sequence from a possible hexaploid hybrid taxon, *C. turcicum*.

## 3. Discussion

### 3.1. About the Sample Set

**Figure 6:**
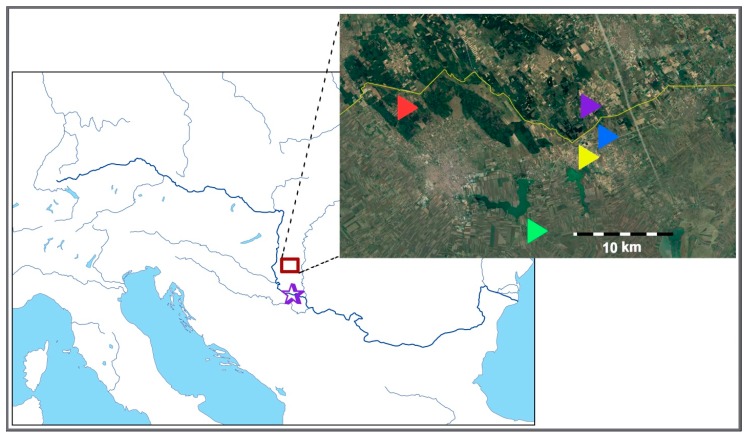
Map of south-east Europe with a detailed terrain map of the study area of Subotica Sands. The colored triangles on the extracted Google map* represent localities of existing populations in the area: red—Majdan (MJ); green—Palić (PA); yellow—Hajdukovo (HA); blue—Bački Vinogradi (BV); purple—Ásotthalom (AS). The purple star on the basic map represents approximate position of Fruška Gora Mt. populations of *C. erythraea*—Iriški venac (IV) and Beočin (BE). * Satellite image downloaded from Google Earth software (Google Earth Pro, v. 7.3.2.5776, recording date: March 31st, 2019, Subotica, view height: 32.33 km, DigitalGlobe 2019, download date: 6 May 2019).

The focus of this research was placed on the interspecific hybridization between two tetraploid species belonging to the genus *Centaurium*, *C. erythraea* and *C. littorale* subsp. *compressum*. Hybrid individuals having a hexaploid genome were only recently noticed in two localities, namely Majdan—**MJ** and Palić—**PA**. The aims and the directions of the presented research have been conceived on the basis of several facts and observations. Firstly, literature data indicate great phylogenetic relatedness of *C. erythraea* and *C. littorale* [8,34,37]. Secondly, examples of hybridization of certain subspecies of these two species have been previously documented and confirmed by various methods [8,11,12,13]. The fact that these authors have reported hybridization between *C. erythraea* and *C. littorale* in geographically remote localities from our study site led us to presume that the hybrids might arise after independent hybridization events, which certainly deserves our attention. Finally, a research on interspecific hybridization would possibly raise public awareness on importance to further protect Subotica Sands and Nature Park Palić, particularly the locations **MJ** and **PA**, since the ubiquitous arable land of these plain landscapes, roads, and other obstacles derived from human activity represent barriers for species distribution and cause strong fragmentation of the habitat (as can be seen on Figure 6). Although disadvantageous for the survival of the species, such relatively small number of clearly restricted fragmented populations provides a good basis to study cross-species hybridization incidence in natural conditions within the genus *Centaurium*.

**Figure 7:**
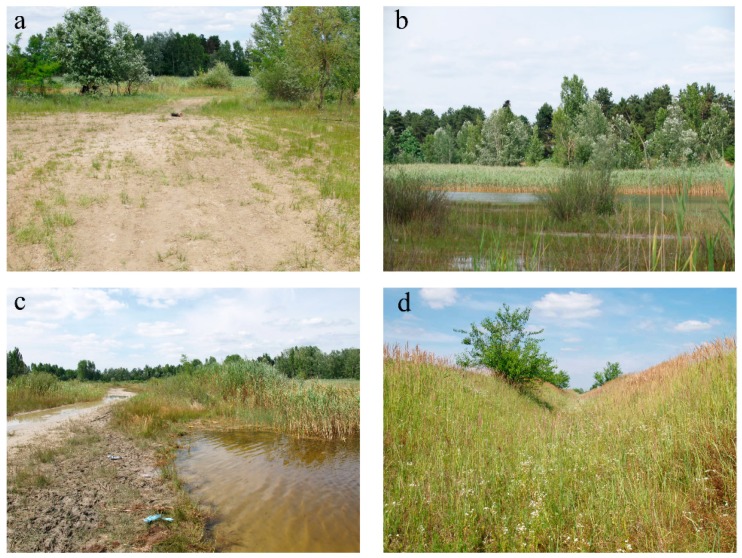
Localities where hexaploid plants characterized as *C. pannonicum* were found. (**a**–**c**)—Majdan (**MJ**); (**d**)—Palić (**PA**).

This study included all four existing populations of *C. littorale* subsp. *compressum* and two of *C. pannonicum* found in the specified area. On the other hand, due to the deficiency of nearby populations of *C. erythraea*, in addition to neighboring **BV**, two populations located about 200 km south, on the Fruška Gora Mountain were included in this research. Plants of *C. littorale* subsp. *compressum* and *C. pannonicum* were found growing in sympatry on the localities **MJ** and **PA** (Figure 6 and Figure 7) but the second species involved in hybridization, *C. erythraea*, was not found there. This fact raises the following question: since growing in sympatry represents the main prerequisite for the interspecific hybridization to occur, particularly for species which are pollinated by short-distance insects [38], why is *C. erythraea* no longer found in these locations? Literature data indicate that *C. erythraea* and *C. littorale* subsp. *compressum* exhibit slightly different habitat preferences: while *C. erythraea* is more often found on dry limestone soils, and rarely in loamy, sandy and wetlands, *C. littorale* subsp. *compressum* has a preference for settled and humid habitats [39]. Also, in the study of interspecific hybridization of *C. erythraea* subsp. *erythraea* and *C. littorale* subsp. *littorale* on the territories of Great Britain, Germany and Denmark, one of the author’s conclusions was that these taxa have shown different habitat preferences [13]. According to this study, the most probable assumption is that the two species step into hybridization when the habitat is disturbed, mostly by human activity, which potentiates their contact. Localities **MJ** and **PA** are certainly habitats created by human activity and it can be reasonably assumed that the emergence of a hybridogenic taxon at these sites was the result of anthropogenic changes. Once established, the hybridogenic taxon can push out one or both parental species destroying the evidence of its own hybrid origin [4,10]. Bearing that in mind, we may hypothesize that this situation has happened in the case of interspecific hybridization between *C. erythraea* and *C. littorale* subsp. *compressum*. *C. erythraea*, as a species that prefers dryer habitats, may have been suppressed by the other two taxa and finally perished from the mutual habitats. Changes in the habitat caused by human activity can greatly alter ecological conditions and thus favor some groups of organisms that were not competitive before the disturbance [1]. Our study points to a stunning postulation that the activities of people, completely unconsciously, by altering life conditions in habitats, may have led to the creation of a new hybridogenic taxon. Although the fate of this taxon in nature may be uncertain, the results of this research point out to the need to protect the unique natural goods of Majdan and Palić, which represent places where it can be possible to follow evolutionary processes, reproductive isolation, and speciation in situ.

### 3.2. Morphometrics in the Detection of Hybrids

A well-known fact is that plant morphological characteristics may be highly susceptible to environmental factors, edaphic conditions, seasons, plant infections, pests, etc. This is especially true for plants belonging to the genus *Centaurium* in which high clinal variation in gross morphology, often caused by ecological conditions, is frequently observed [7,13,16,24,34]. However, meticulous selection of parameters to consider for a study, which would be only negligibly influenced by environmental pressures, is of critical importance to obtain an unbiased morphometric comparison. The second challenge of this part of the study was to select morphological traits which would have enough resolution power to differentiate between two closely related species and the hybridogenic taxon. For instance, as proposed by Ubsdell [11], we have studied pollen grains’ dimensions of the three taxa, determined to find the differentiation among them. However, we recorded variance that was several times greater within the taxa than among them and had to discard this parameter as uninformative.

As can be seen from Figure 1 and Figure 2, as well from Figure S1, five selected morphological traits showed different potential to support differentiation between the taxa. For some characteristics, basic statistics indicated that they were under higher environmental pressures than others. This specifically refers to length/width ratio of floral stalk leaves. It can be observed (Figure S2) that the two populations of *C. pannonicum* considerably differ among themselves regarding this trait, which can be explained by the different habitats the plants grew in. Plants from the population **MJ** were found in an open habitat with only a few other plant species found in the same phytocenosis (Figure 7a–c). They grew up to 30 cm in height and have developed smaller leaves due to high insolation. Contrastingly, plants from the population **PA** were found in an irrigation canal in a semi-shaded habitat where the plant community consisted of at least twenty different, densely growing species (personal observation, Figure 7d). In this habitat, *C. pannonicum* developed several times longer leaves on plants up to 80 cm tall (Figure S3). Leaf width, however, was mildly affected by such an environment, which resulted in more lanceolate leaves (Figure S4). Therefore, while highly appreciated to differentiate two parental species, length/width ratio of floral stalk leaves showed weak relevance in delimitation of interspecific hybrids, as proposed by Ubsdell [11], although this trait, along with leaf tip angle, falls among the indispensable basic morphological key parameters for use in the field studies for the determination of the potential hybrid plants (personal observation). As the results of such practice applied in the field, in the whole study we failed to correctly determine only one plant (*C.ery.***BV** 3190).

Data obtained by measuring other parameters, such as calyx/corolla-tube ratio and length of corolla lobes, were highly concordant with previous studies [11]. The calyx/corolla-tube ratio delimitates all the three taxa quite well, while the length of corolla lobes gives a good basis for discrimination of sympatric taxa *C. littorale* subsp. *compressum* and *C. pannonicum* (Figure S1).

In the PCA biplot based on the five morphometric traits (Figure 1) we can notice partial overlap of the hybrid individuals’ cloud with the clouds of both of the parental species. This could explain why this hybridogenic taxon has remained unnoticed until few years ago, as well as why sporadic misidentifications occur in the field during the collection of plant material while opening possibilities for disambiguation in taxonomy of the genus [24,34].

Although we were guided by previous morphological analyses [11] in the initial selection of morphological traits to be measured, it was not possible to give a diagnostic key which would avoid overlapping in the character measures of the hybrid and either of the parental species. It may be necessary to emphasize once again that the two parental species are very closely related and such a situation regarding the lack of clear differences in the morphological characteristics is highly anticipated.

More thorough studies, which would include additional morphological and anatomical characters, might give a clearer delimitation of *C. pannonicum* from *C. erythraea* and *C. littorale* subsp. *compressum*.

### 3.3. Genetic Variation

To estimate genetic variation in natural populations of both parental species and the interspecific hybrid, the EST-SSR multilocus genotyping was performed and reported for the first time for the populations within the genus *Centaurium*. For this purpose, we employed newly designed genic microsatellite markers [36]. 

The specificity of the microsatellite markers enabled us to analyze plant material collected directly from nature, without amplifying foreign (e.g., fungal or insect) DNA, which may give false variations when non-specific markers are used. In the previous study [36], we confirmed the transferability of the eight genic microsatellite markers, initially developed for *C. erythraea*, to seven other taxa, and, among them, the two studied here. Still, since EST-SSR markers are basically developed from *C. erythraea* transcriptome, it is not surprising that they showed higher variability in this species (Table 1) in comparison to the other two studied taxa [40,41].

The most prominent genetic variability within the *C. erythraea* populations was recorded for Bački Vinogradi (**BV**). This might happen because **BV** locality may be from time to time populated with hybrid individuals and also with *C. littorale* subsp. *compressum*, a few of them spotted in 2018, which certainly promotes the gene flow between the taxa. This may be the main reason for misidentification of the individual *C.ery.***BV** 3190, which turned out to group with *C. pannonicum* (Figure 3). Plastid DNA marker analysis supports this conclusion (please see below). However, our previous research on populations’ ploidy [32] did not reveal hexaploid individuals in this locality. Does this indicate continuous gene flow between different tetraploid taxa? Probably yes. Similar examples have been reported earlier [8,13,24]. This information can give very good support to the hypothesis that hybridization between *C. erythraea* and *C. littorale* subsp. *compressum* is very common and may or may not include polyploidization. The mentioned individual, *C.ery*.**BV** 3190, is also positioned somewhere on the edge of the *C. erythraea* cloud in the PCA scatterplot based on morphometrics (Figure 1, marked with a rectangle around the corresponding point). LDA based on morphometrics data did not include this individual. 

Within the *Gentianaceae* family, sequences of the non-coding *trn*L (UAA) intron and *trn*L-*trn*F spacer region from the plastid DNA have been previously analyzed in order to estimate phylogenetic relationships [34,42,43,44,45], or to detect evidence of reticulation [8,24]. The primer combination used for the amplification of this plastid non-coding region (“c” and “f” primers reported by Taberlet et al. [46]) was not used in similar studies. Such a combination was used here in order to provide longer amplicons which would consist of two joint plastid regions, *trn*L (UAA) intron and *trn*L-*trn*F spacer region. Nevertheless, within this research, it was not possible to read the sequences with primer “c”, probably due to the existence of polyA region (about 17–20 bp), which was observed on the forward part of the sequences. PolyA regions are unstable, causing polymerase slips during extension, and thus making sequencing impossible [47]. Because of this inconvenience, the sequences obtained in this study were aligned only according to intergenic spacer *trn*L-*trn*F sequences of closely related taxa and potential hybrid individuals retrieved from GenBank. Although the resulting matrix of the aligned sequences consisted of just 380 characters, the phylogenetic analysis showed two distinctive haplotypes present among all analyzed individuals. The same haplotypes and affiliations were recorded with 43 longer sequences (504–514 bp), which were obtained by sequencing the amplicons in only one direction (results are not shown but are available upon request). 

Plastid DNA is maternally inherited in the genus *Centaurium* [10,48] and the analysis of plastid haplotypes was done in order to reveal the maternal progenitor of the hybridogenic taxon *C. pannonicum*. Plastid introgression after intrageneric interspecific hybridization is a well-documented event [49,50]. The analysis of the plastid haplotypes reported here, clearly indicates that the most likely plastid donor (and therefore the maternal species) to the hybridogenic taxon *C. pannonicum* were some population(s) of *C. littorale* subsp. *compressum* from the same or nearby areas, since all analyzed individuals of *C. pannonicum*, as well as all individuals of *C. littorale* subsp. *compressum*, have been shown to possess the same plastid haplotype. Three individuals of *C. erythraea* share the same haplotype but these individuals belong to distant populations (**BE** and **IV**; Figure 6). An interesting fact is that one individual of *C. erythraea* from Bački Vinogradi (*C.ery.***BV** 3190) also showed a haplotype characteristic for hybridogenic taxon and *C. littorale* subsp. *compressum*. However, for this individual, with very high certainty, we can infer it was incorrectly determined. As already mentioned, the analysis of microsatellite markers also grouped this individual with *C. pannonicum*. In general, two haplotypes can be noticed within the sample set made up from the studied individuals and previously reported GenBank sequences, but in some cases, it was not possible to link haplotypes to a particular species. It can be rather said that different haplotypes could be linked to different populations. This claim can be supported by the repeatedly underlined (and studied) fact that interspecific hybridization within the genus *Centaurium* occurs very commonly [7,10,13,16,51,52], leading to reticulate evolution of the plastid haplotypes. Because of its haploid nature and mode of inheritance, plastid genome is more sensitive to genetic drift effects, which may result in fixation of population-characteristic haplotypes. 

When talking about the direction of interspecific hybridization, the choice of which species would act as the pollen donor may be influenced by various ecological factors (e.g., the number of individuals of a particular species in the habitat, pollinators’ preferences), as well as the biological characteristics of two species which hybridize (e.g., pollination mechanisms, flowering period, different habitat preferences). In his extensive studies on hybridization between *C. erythraea* subsp. *erythraea* and *C. littorale* subsp. *littorale*, Ubsdell [11,12,13] has come to a conclusion that the pollen donor had to be *C. littorale* subsp. *littorale* individuals due to slightly different flowering period and difference in maturity of stigmas and pollen grains between the two taxa. On the other hand, when we “switched off” the ecological factors and the species-specific biology by performing artificial hybridization under in vitro conditions [9], seeds were formed only in cases when the pollen donor was *C. erythraea*. 

Among the populations studied here, the analysis with microsatellite molecular markers showed closer phylogenetic relationship of hybridogenic taxon *C. pannonicum* with *C. erythraea* than with *C. littorale* subsp. *compressum*. On the contrary, plastid *trn*L-F marker haplotyping pointed at *C. littorale* subsp. *compressum* to be the maternal species of *C. pannonicum*. The similar situation of discordant data obtained with differently inherited markers is often found among plant species [8,53]. Additional screening with differently inherited markers, possibly with other DNA barcoding markers, could provide more information and enrich our perspective on this amazing phenomenon. 

### 3.4. Origin of Centaurium pannonicum

**Scheme 1:**
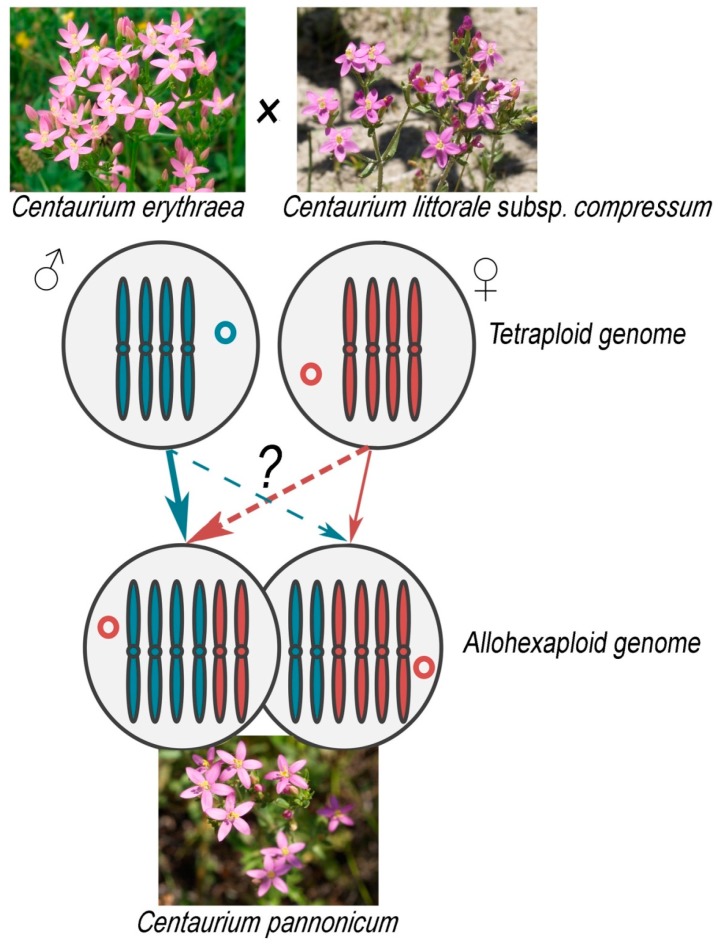
Proposed model for the formation of allohexaploid hybrids between tetraploid taxa *C. littorale* subsp. *compressum* and *C. erythraea*. Dashed arrows represent reduced, while full-line arrows represent non-reduced gametes that participate in the interspecific hybridization event. Plastid DNA is presented as either red or blue circle.

As previously mentioned, the basic aim of this study was to set up the genetic and morphological frame for understanding and characterizing a specific case of the interspecific hybridization within the genus *Centaurium*. Our earlier study [32] had already established a solid basis to fathom this evolutionary mechanism when it comes to examine chemodiversity, as well as the difference in the ploidy levels of parental and hybridogenic taxa. The present study, which involved both morphometrics and different types of molecular markers, along with the previous research, strongly supports the hypothesis of allopolyploid origin for *C. pannonicum* at two locations (**PA** and **MJ**) and determines with high confidence its tetraploid progenitors (Scheme 1). 

While the EST-SSR genotyping indicates closer genetic relationship of *C. erythraea* and *C. pannonicum*, sharing the same plastid marker haplotype by all the individuals of *C. pannonicum* and *C. littorale* subsp. *compressum* undoubtedly nominee *C. littorale* subsp. *compressum* as the other taxon that has participated in this interspecific hybridization process, the same being supported by the results of the phytochemical profiling [32]. Moreover, the results of the analysis of *trn*L-F region of the plastid DNA led us to propose the tetraploid taxon *C. littorale* subsp. *compressum* as the plastid donor (maternal progenitor) of the hexaploid hybrid *C. pannonicum*, and consequently, the tetraploid taxon *C. erythraea* as the pollen donor (Scheme 1). Nevertheless, it still remains to answer the question as to which of the two species involved in the process of interspecific hybridization contributed to the hexaploid *C. pannonicum* genome with four chromosomal sets and which with two. The closer relationship with *C. erythraea* revealed by EST-SSR genotyping implies that this parental species might contribute with four chromosomal sets to the hexaploid hybrid genome (bold arrows in Scheme 1). This issue may open up the opportunities for future research which would include cytogenetic methods of counting, identification and differential staining of chromosomes, FISH (Fluorescent In Situ Hybridization) and GISH (Genomic In Situ Hybridization) methods that could return the final solution about the share of parental genomes in hybridogenic taxon [54,55]. 

## 4. Materials and Methods 

### 4.1. Sampling Sites

Plant materials were collected at the localities presented in Table 3 during June 2015 in the Subotica Sands, around Lake Palić and Fruška Gora Mt. (Vojvodina, Serbia) (Figure 6 and Figure 7). Subotica Sands are protected area with sustainable use of natural resources (category VI according to IUCN), Nature Park Palić is classified as protected landscape (IUCN category V) while National Park Fruška Gora is classified in category II according to IUCN. Plants were identified in the field by the authors (B. Šiler, T. Banjanac and D. Mišić)-based either on available taxonomic keys [24] in the case of *C. erythraea* and *C. littorale* subsp. *compressum* or solely on morphological characters of habitus in the case of individuals classified as *C. pannonicum*. Their taxonomical status was investigated earlier by both flow cytometry and phytochemical profiling [32]. Ploidy levels of the collected plants have been previously published [32] and are given in Table 3. Corresponding voucher specimens were deposited in the herbarium of the University of Belgrade, Serbia (BEOU, Thiers, 2019; voucher numbers: 17271–17279).

### 4.2. Morphometric Analysis

#### 4.2.1. Plant Material

A set of 127 individuals belonging to the three *Centaurium* taxa was included in the morphometric analyses. Immediately after plant harvesting, three randomly chosen flowers and the first three leaves above the rosette per plant were collected and preserved in a mixture of 35% ethanol and 20% glycerol (v:v = 3:1) for the comparative study of morphological characters (Figure S5). Samples were kept at room temperature until use.

#### 4.2.2. Analysis of Morphological Traits

After careful consideration of the morphological characters suggested by Ubsdell [11] and the basic statistical analysis of the morphological parameters (Figure S1), six leaf and flower quantitative traits were selected, measured using Digimizer Image Analysis software (MedCalc Software, Belgium) and presented as the following characteristics: length/width ratio of floral stalk leaves, calyx/corolla-tube ratio, length of corolla lobes, and leaf tip angle which was decomposed to the sine and cosine components. Length/width ratio of floral stalk leaves was obtained by dividing the total length of a leaf by its greatest width. For each plant, the first three floral stalk leaves above the basal rosette were measured and a mean value was obtained. Calyx/corolla-tube ratio was obtained by dividing the total length of the calyx from the base to the tip of the teeth by the length of the corolla-tube. Length of corolla lobes was measured from the top of the corolla-tube to the tip of the corolla lobes. Three flowers per plant were scored and mean values were obtained (Table S2). 

#### 4.2.3. Statistical Analysis of the Morphometric Data

Morphometric data were analyzed with both principal component analysis (PCA) and linear discriminant analysis (LDA) using the R programming language packages ‘stats’, ‘MASS’ [56] and ‘caret’ [57]. PCA was performed on zero centered and unit scaled data. To evaluate the number of components that should be retained in PCA, Horn’s parallel analysis [58] was performed using the R package ‘paran’ [59]. To assess the usability of the quantitative characters in predictive classification, LDA was assessed with five times repeated five-fold cross-validation using prediction accuracy on the hold out instances as metric. Furthermore, LDA was performed with a presumption of equal prior probability of classes. Feature selection for LDA was performed using a wrapper approach by sequential backwards search as implemented in ‘caret’ package.

### 4.3. EST-SSR Genotyping

#### 4.3.1. Plant Material for the DNA Extraction

For the purposes of DNA extraction, young leaves were collected from the upper half of the flower stem and immediately stored in plastic zip-bags containing silica gel. Samples were kept in darkness, at the room temperature until use. In total, 136 individual plants were included in the ESR-SSR genotyping (Table 3).

#### 4.3.2. DNA Isolation and EST-SSR Amplification

Total genomic DNA was isolated from dried leaf samples (20 mg leaf tissue) using a modified CTAB method [60]. DNA concentration and the purity of the isolates were assessed by the spectrophotometric absorbance at 260, 280, and 230 nm (Agilent 8453, Agilent Technologies, Waldbronn, Germany). 

Primer pairs (Invitrogen, Thermo Fisher Scientific, Carlsbad, CA, USA) for amplification of eight EST-SSR loci (M4, M5, M7, M8, M10, M12, M13 and M17), previously published by Banjanac et al. [36], were employed to study the genetic variation within the sample set. PCR amplifications were conducted according to the same study as well. Fragment analysis of PCR products was performed using the Lab-on-a-Chip technology (DNA 1000 LabChip) on the Agilent Bioanalyzer 2100 system (Agilent Technologies, Santa Clara, CA, USA). PCR products’ peaks on electropherograms were aligned using Agilent 2100 Expert software (Agilent Technologies, Santa Clara, CA, USA) as described in Banjanac et al. [36]. Alleles were scored manually. 

#### 4.3.3. Genetic Diversity of Populations Based on the EST-SSR Variation

Data obtained by eight EST-SSR molecular markers of both tetraploid and hexaploid plants were first analyzed using the package ‘polysat’ [61] in R programming language [62]. Distances between genotypes were calculated by the method described by Bruvo et al. [63] using the ’meandistance.matrix2’ function in ‘polysat’ with ‘Bruvo2.distance’ distance metrics (equation 6 in [63]). The basic settings within the ‘polysat’ package included presumptions such as: polysomatic way of inheritance for all the populations, corresponding ploidy of populations (tetraploid/hexaploid, according to Banjanac et al. [32]), selfing rate of 0.56 (according to Brys and Jacquemyn [38]) and the length of microsatellite repeats for each locus (see in [36]). The genetic variability was estimated using the following parameters: the total number of alleles in a population, the average number of alleles per locus per population, the observed (H_O_) and the expected heterozygosity (H_E_), and the effective number of alleles in a population based on the expected heterozygosity. H_O_ of a population was obtained by counting heterozygous individuals, while H_E_ was calculated on the basis of allele frequencies generated by ‘polysat’ using formula:

H_E_ = 1 − Σpi^4^ for tetraploid populations and

H_E_ = 1 − Σpi^6^ for hexaploid populations,

where p is the frequency of the i^th^ allele of a given locus. 

Given genetic data, allele frequencies by population were calculated using the ‘simpleFreq’ function in ‘polysat’ package for R, assuming polysomic inheritance. In the following step, using the data frame of allele frequencies and population sizes, we calculated a matrix of pairwise F_ST_ and Jost’s D values using the function ‘calcPopDiff’ of the same R package.

#### 4.3.4. Visualization of the Genetic Distances

The obtained Bruvo genetic distances among the individuals were visualized using two-dimensional nonmetric multidimensional scaling (NMDS) which was performed employing the package ‘vegan’ for R [64].

Further representation of relationships within the sample set included hierarchical cluster analysis (HCA), using UPGMA method. The dendrogram was constructed on the basis of the Bruvo genetic distances between individuals, which were obtained from the allele frequencies at eight EST-SSR loci, calculated by ‘polysat’ package. Assessment of the clusterwise stability of the clustering was performed by bootstrapping using the R package ‘fpc’ [65].

### 4.4. trnL-F Haplotype Analysis

#### 4.4.1. Sample Set

A subset of 43 DNA isolates (Table 3) was used to assess the variation in the plastid genome through *trn*L-F haplotyping. Analysis of the variation in plastid *trn*L-F region also included 16 GenBank accessions of the taxa of interest previously reported by other authors (Table 4). 

#### 4.4.2. Plastid DNA Amplification and Marker Sequencing

Two non-coding regions of plastid DNA, *trn*L UAA intron and the *trn*L-*trn*F intergenic spacer were amplified in the same reaction using primers reported by Taberlet et al. [46]: “f” (ATT TGA ACT GGT GAC ACG AG) and “c” (CGA AAT CGG TAG ACG CTA CG). PCR amplifications were carried out using Eppendorf Mastercycler nexus gradient thermal cycler (Eppendorf AG, Hamburg, Germany) in a final volume of 25 µl. Each reaction contained 100 ng of the template DNA, DreamTaq Green PCR Master Mix (according to the manufacturer’s instructions; Thermo Fisher Scientific, Waltham, MA, USA) and 0.5 µM of both forward and reverse primers (Invitrogen^®^, Thermo Fisher Scientific, Carlsbad, CA, USA). The PCR reaction program used for the amplification of this combined plastid non-coding regions was as follows: 94 °C for 10 min; 35 cycles of 94 °C for 1 min, 55 °C for 45 s, 72 °C for 1.5 min, and 72 °C for 10 min as a final extension step.

Amplified PCR products were purified using off-the-shelf absorption, washing and elution DNA buffers, and EconoSpin DNA spin columns according to the manufacturer’s instructions (http://www.epochlifescience.com/). They were run on a 1% agarose gel stained with ethidium bromide, in order to evaluate the quality and quantity of the amplified templates.

Direct sequencing was performed on an ABI 3130 Genetic Analyzer (Applied Biosystems, Thermo Fisher Scientific, Waltham, MA, USA), using BigDye Terminator v. 3.1 Cycle Sequencing Kit (Applied Biosystems, Thermo Fisher Scientific, Waltham, MA, USA) for cycle sequencing reactions following the manufacturer’s instructions. All samples were sequenced with “f” primer. The obtained results were visualized and analyzed using the Sequencing Analysis Software 6 (Applied Biosystems, Thermo Fisher Scientific, Waltham, MA, USA) and BioEdit (Ibis Biosciences, Carlsbad, CA, USA). Sequences alignment was done with MUSCLE program (https://www.ebi.ac.uk/Tools/msa/muscle/). Selection of conserved blocks from the alignment was performed using Gblocks v0.91b [66] with the following options: minimum number of sequences for a conserved position was set to 30, minimum number of sequences for a flank position was set to 50, maximum number of contiguous non-conserved positions was set to 8, minimum length of a block was set to 10, while the allowed gap positions were set to half.

The resulting alignment was visualized with FastTree v2.1.10 [67] using GTR model (generalized time-reversible model [68]) of evolution. Phylogenetic tree was rooted by *Centaurium pulchellum*. Local support values were estimated using the Shimodaira-Hasegawa test on the three alternate topologies around the specific split [67]. A total of 43 sequences were generated and submitted to GenBank (accession numbers MN104899–MN104941).

## 5. Conclusions

Plenty of evidence reveals the importance of both polyploidization and interspecific hybridization as evolutionary mechanisms responsible for speciation within the genus *Centaurium* [8,10,34,51]. We hope that the results of the presented research will contribute to the clarification of the phylogenetic relationships within the genus but also to the understanding of the processes of polyploidization and interspecific hybridization and their roles in comprehending the concept of plant species. In the presented case, we are witnessing the generation of evolutionary novelty and the promotion of expansion of the allopolyploid hybridogenic taxon, possibly endowed with new or improved adaptive characteristics, which might increase its evolutionary potential. This is extremely important if we consider rapid climate changes, but also severe anthropogenic intrusion in most of the known *C. pannonicum* habitats. In the end, but certainly not less important, is the unavoidable need to design and suggest a conservation strategy for new hybridogenic taxon *C. pannonicum* by evaluating its genetic diversity and the evolutionary potential of parental species in the zones where the hybrids may arise, as well as to define the current status of the habitats and to recognize possible threats. As a means of ex situ conservation strategy, we have established a seed collection representing the genetic diversity of *C. pannonicum* in its natural populations, which will enable the long-term preservation of its germplasm. On the other hand, as a means of in situ conservation, we may propose raising the level of protection of the sites and the prohibition of further habitat disturbance by humans.

## Figures and Tables

**Table 1:** Population genetics parameters obtained by eight genic microsatellite loci: the total, average and effective number of alleles in populations and the values of the observed (H_O_) and expected heterozygosity (H_E_) of the populations are presented.

Populations	Number of Analyzed Individuals per Population	Total Number of Alleles in a Population (all 8 loci)	Average Number of Alleles for All Loci	Effective Number of Alleles in a Population	Average Expected Heterozygosity per Population (H_E_)	Average Observed Heterozygosity per Population (H_O_)
*C.ery.* **BE**	17	29	3.6250	1.1400	0.8772	0.5588
*C.ery.* **IV**	19	31	3.8750	1.1326	0.8829	0.5658
*C.ery.* **BV**	20	34	4.2500	1.1198	0.8930	0.5938
*C.pan.* **MJ**	7	31	3.8750	1.0089	0.9912	0.7969
*C.pan.* **PA**	22	33	4.1250	1.0114	0.9888	0.7337
*C.l.c.* **MJ**	13	20	2.5000	1.1303	0.8847	0.6635
*C.l.c.* **PA**	5	20	2.5000	1.1482	0.8709	0.5750
*C.l.c.* **HA**	15	24	3.0000	1.0995	0.9095	0.7750
*C.l.c.* **AS**	18	24	3.0000	1.0982	0.9105	0.8125
total	136					
average		27.33	3.4167	1.0988	0.9121	0.6750

**Table 2:** Estimated genetic differentiation of populations based on F_ST_ and Jost’s D indices.

	Jost’s D	*C.ery.* **IV**	*C.ery.* **BE**	*C.ery.* **BV**	*C.pan.* **MJ**	*C.pan.* **PA**	*C.l.c.* **HA**	*C.l.c.* **MJ**	*C.l.c.* **PA**	*C.l.c.* **AS**
F_ST_										
*C.ery.* **IV**			0.015	0.055	0.106	0.113	0.200	0.241	0.173	0.210
*C.ery.* **BE**		0.019		0.063	0.111	0.108	0.200	0.240	0.180	0.208
*C.ery.* **BV**		0.126	0.141		0.072	0.096	0.144	0.174	0.114	0.158
*C.pan.* **MJ**		0.282	0.270	0.236		0.031	0.173	0.210	0.170	0.184
*C.pan.* **PA**		0.305	0.300	0.312	0.124		0.154	0.162	0.090	0.179
*C.l.c.* **HA**		0.526	0.497	0.433	0.553	0.514		0.033	0.024	0.013
*C.l.c.* **MJ**		0.553	0.509	0.437	0.547	0.501	0.054		0.007	0.032
*C.l.c.* **PA**		0.533	0.491	0.415	0.507	0.467	0.039	-0.021		0.030
*C.l.c.* **AS**		0.552	0.519	0.466	0.609	0.573	0.019	0.065	0.077	

**Table 3:** *Centaurium erythraea*, *C. littorale* subsp. *compressum* and *C. pannonicum* accessions used in the study.

Taxon	Locality	Population Accession Code	Geographic Latitude and Longitude	Estimated Ploidy Level *	No. of Studied Individuals		
					EST-SSR	Morphometrics	Plastid DNA
*C. erythraea*	Beočin	*C.ery*.**BE**	45°10′35.24″ N 19°43′16.32″ E	4x	17	17	2
*C. erythraea*	Iriški Venac	*C.ery*.**IV**	45°09′27.65″ N 19°51′43.64″ E	4x	19	19	3
*C. erythraea*	Bački Vinogradi	*C.ery.* **BV**	46°07′29.19″ N 19°50′56.02″ E	4x	20	20	10
*C. littorale* subsp. *compressum*	Majdan	*C.l.c*.**MJ**	46°09′29.34″ N 19°36′20.53″ E	4x	13	13	4
*C. littorale* subsp. *compressum*	Palić	*C.l.c*.**PA**	46°03′08.04″ N 19°46′34.89″ E	4x	5	4	3
*C. littorale* subsp. *compressum*	Hajdukovo	*C.l.c*.**HA**	46°06′52.27″ N 19°49′53.58″ E	4x	15	15	4
*C. littorale* subsp. *compressum*	Ásotthalom	*C.l.c.* **AS**	46°11′04.50″ N 19°50′47.62″ E	4x	18	18	1
*C. pannonicum*	Majdan	*C.pan.* **MJ**	46°09′29.34″ N 19°36′20.53″ E	6x	7	5	5
*C. pannonicum*	Palić	*C.pan*.**PA**	46°03′08.04″ N 19°46′34.89″ E	6x	22	16	10
*C. pulchellum*	Palić	*C.pul.* **PA**	46°03′08.04″ N 19°46′34.89″ E	NA	/	/	1
in total					136	127	43

* Based on the results published in Banjanac et al. [32].

**Table 4:** Origin of plant material, reference information and GenBank accessions for DNA sequences used for comparison of *trn*L-F haplotypes.

Taxon	GenBank Accession No.	Accession Code Used in Manuscript	References	Ploidy Level	Country, Region, Locality
*Centaurium erythraea*	AY315697	*C.ery*.**NA**	Mansion, G. and Zeltner, L.; Unpublished	NA	NA
*Centaurium erythraea*	AY879973	*C.ery*.**FR**	[8]	4x	France, Gard, Espiguette
*C. erythraea × C. littorale*	AY879975	*C.e.*×*C.l*.**DK1**	[8]	4x	Denmark, Jutland, Sand Milen
*C. erythraea × C. littorale*	AY879976	*C.e.*×*C.l*.**DK2**	[8]	4x	Denmark, Jutland, Skallingen
*C. erythraea × C. littorale*	AY879977	*C.e.*×*C.l*.**DK1**	[8]	4x	Denmark, Jutland, Sand Milen
*C. erythraea × C. littorale*	AY879978	*C.e.*×*C.l*.**DK1**	[8]	4x	Denmark, Jutland, Sand Milen
*Centaurium littorale*	AY879979	*C.lit*.**DK3**	[8]	4x	Denmark, Jutland, Grenen
*Centaurium littorale*	AY879980	*C.lit*.**DK4**	[8]	4x	Danemark, Lakolk, Romo Island
*Centaurium littorale*	AY879981	*C.lit*.**DK2**	[8]	4x	Denmark, Jutland, Skallingen
*Centaurium littorale*	AY879982	*C.lit*.**DK2**	[8]	4x	Denmark, Jutland, Skallingen
*Centaurium littorale*	AY251756	*C.lit.* **GB**	[34]	4x	England, Holy Island
*Centaurium turcicum*	AY880009	*C.tur.* **GE**	[8]	6x	Georgia, Agara
*Centaurium turcicum*	AY880010	*C.tur*.**TR1**	[8]	6x	Turkey, Phrygie, Pamukkale
*Centaurium turcicum*	AY880011	*C.tur*.**TR2**	[8]	6x	Turkey, Phrygie, Beysehir
*Centaurium turcicum*	AY880012	*C.tur*.**TR3**	[8]	6x	Turkey, Pamphylie, Aksu Isparta
*C. littorale* subsp. *uliginosum*	AY880013	*C.l.u.* **SE**	[8]	4x	Sweden, Gotland, Herrvik

## Supplementary Materials

The following are available online at https://www.mdpi.com/2223-7747/8/7/224/s1, Table S1: Confusion matrix depicting the performance of the LDA model in taxon discrimination. The model was constructed using the following morphometric features: length/width ratio of floral stalk leaves, calyx/corolla-tube ratio, length of corolla lobes, and cosine of the leaf tip angle. Performance was estimated based on the hold out set predictions in 10 times repeated 5-fold cross validation (entries are presented as percent average cell counts across resamples), Table S2: Raw morphometric data. Figure S1: Box-plot diagrams depicting distributions of measurements of morphological traits in three taxa of interest. Measurements are in mm except for leaf veins number (discontinuous values 3 or 5) and leaf tip angle which are in degrees of angle. Figure S2: Box-plot diagrams depicting distributions of length/width ratio of floral stalk leaves of *C. littorale* subsp. *compressum* and *C. pannonicum* on two localities (**MJ** and **PA**). Measurements are in mm, Figure S3: Box-plot diagrams depicting distributions of length of floral stalk leaves of *C. littorale* subsp. *compressum* and *C. pannonicum* on two localities (**MJ** and **PA**). Measurements are in mm. Figure S4: Box-plot diagrams depicting distributions of width of floral stalk leaves of *C. littorale* subsp. *compressum* and *C. pannonicum* on two localities (**MJ** and **PA**). Measurements are in mm. Figure S5: Representative photographs of analyzed leaves and flowers belonging to the three studied taxa.
