# Supplementary material for: Phenotypic and Genetic Variation of an Interspecific Centaurium Hybrid (Gentianaceae) and Its Parental Species
Source: Plants (Basel). 2019 Jul 14;8(7):224. doi: 10.3390/plants8070224 (PMC6681202; doi:10.3390/plants8070224)
Supplement: Supplementary file 1 [file plants-08-00224-s001.zip › Supplementary material.docx]

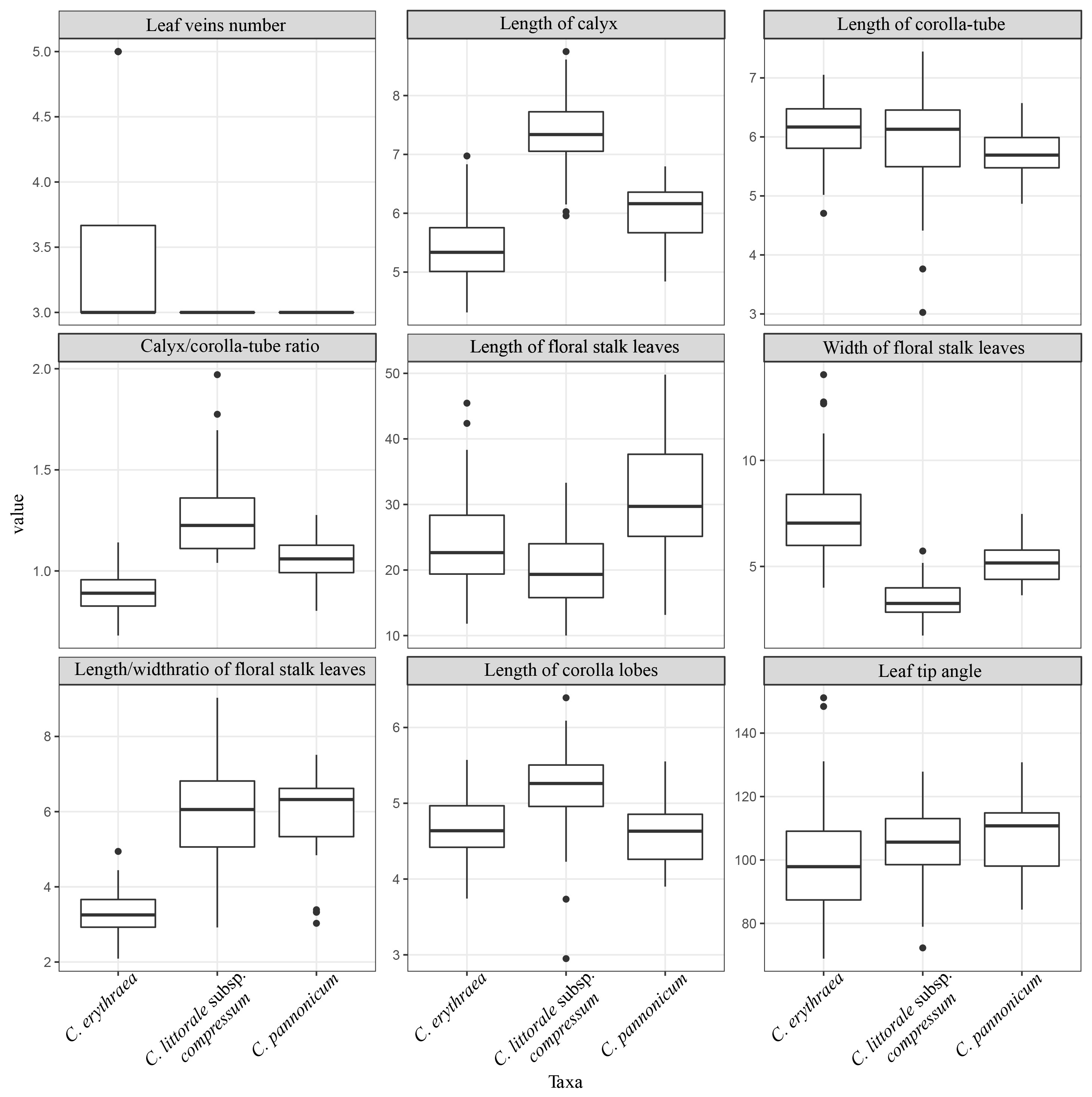


**Figure S1.** Box-plot diagrams depicting distributions of measurements of morphological traits in three taxa of interest. Measurements are in mm except for leaf veins number (discontinuous values 3 or 5) and leaf tip angle which are in degrees of angle.


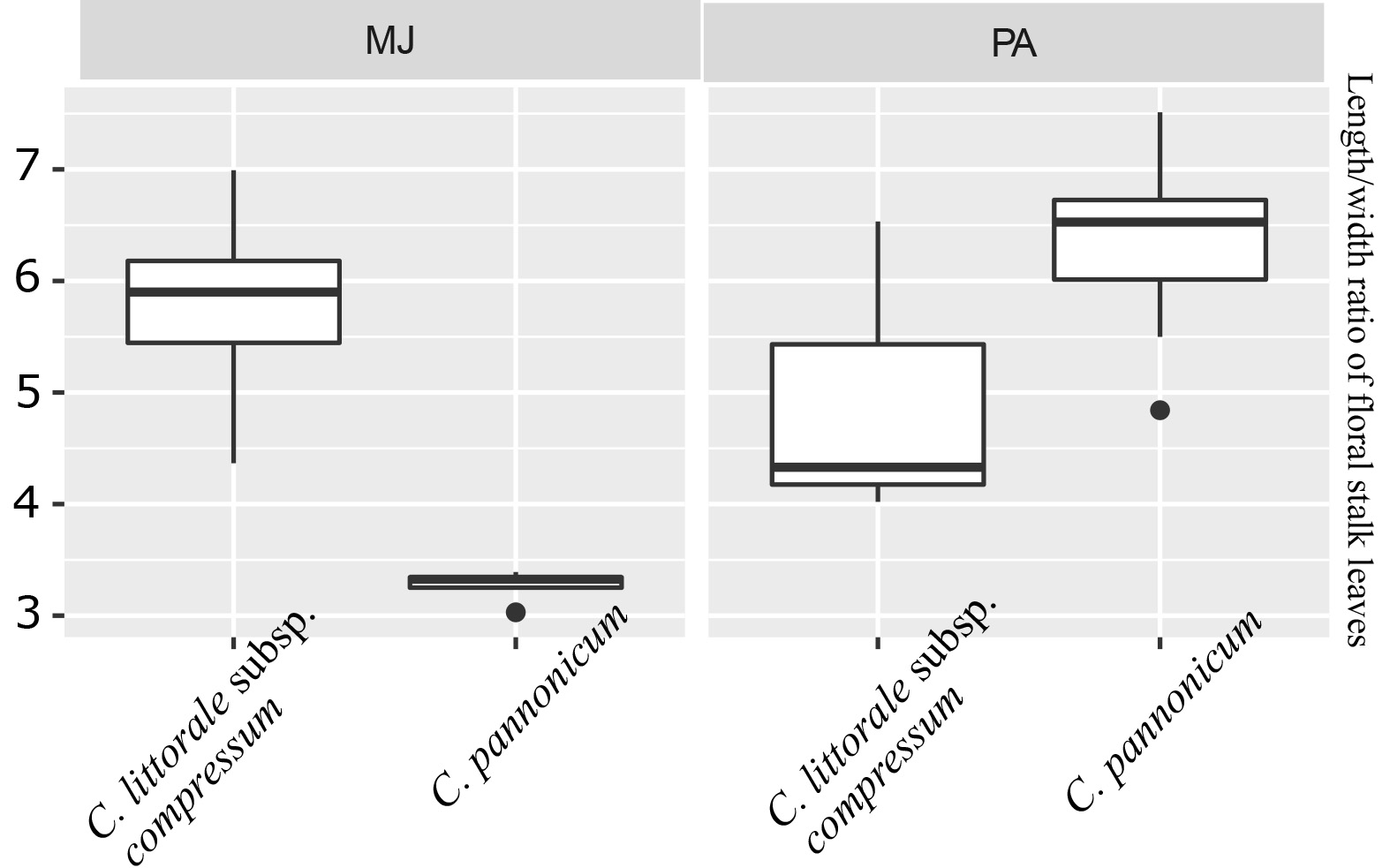


**Figure S2.** Box-plot diagrams depicting distributions of length/width ratio of floral stalk leaves of *C. littorale* subsp. *compressum* and *C. pannonicum* on two localities (**MJ** and **PA**). Measurements are in mm.


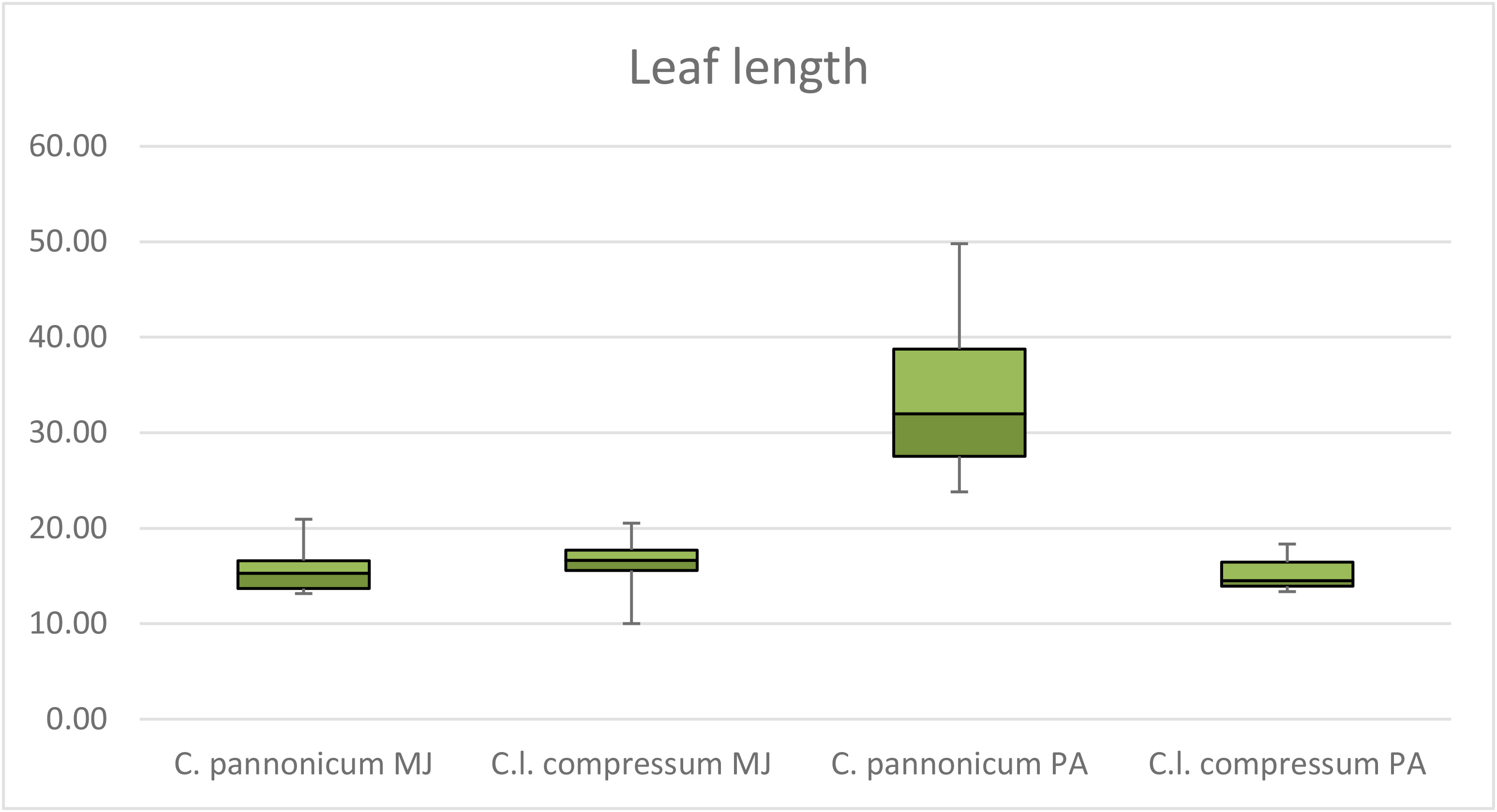


**Figure S3.** Box-plot diagrams depicting distributions of length of floral stalk leaves of *C. littorale* subsp. *compressum* and *C. pannonicum* on two localities (**MJ** and **PA**). Measurements are in mm.


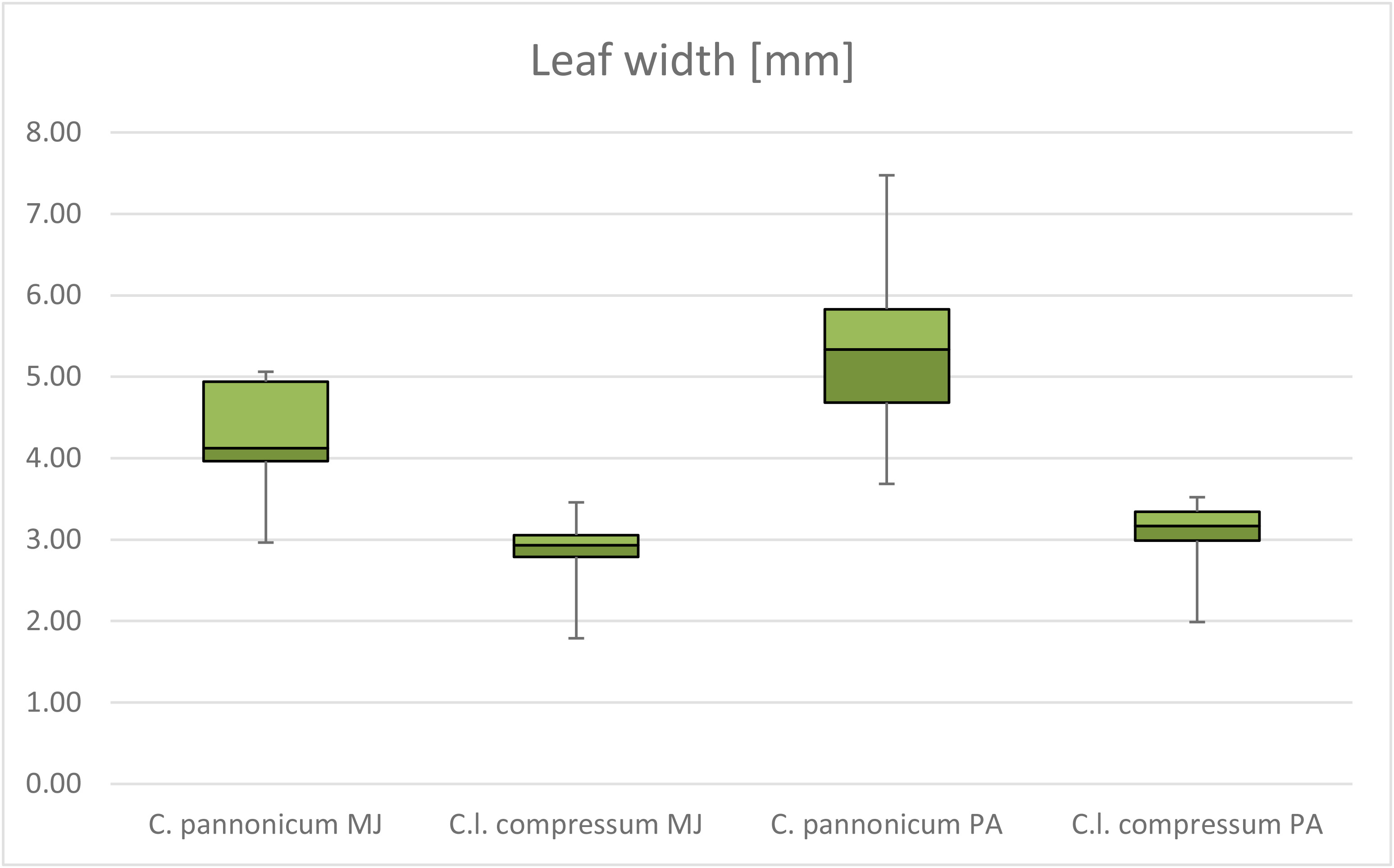


**Figure S4.** Box-plot diagrams depicting distributions of width of floral stalk leaves of *C. littorale* subsp. *compressum* and *C. pannonicum* on two localities (**MJ** and **PA**). Measurements are in mm.


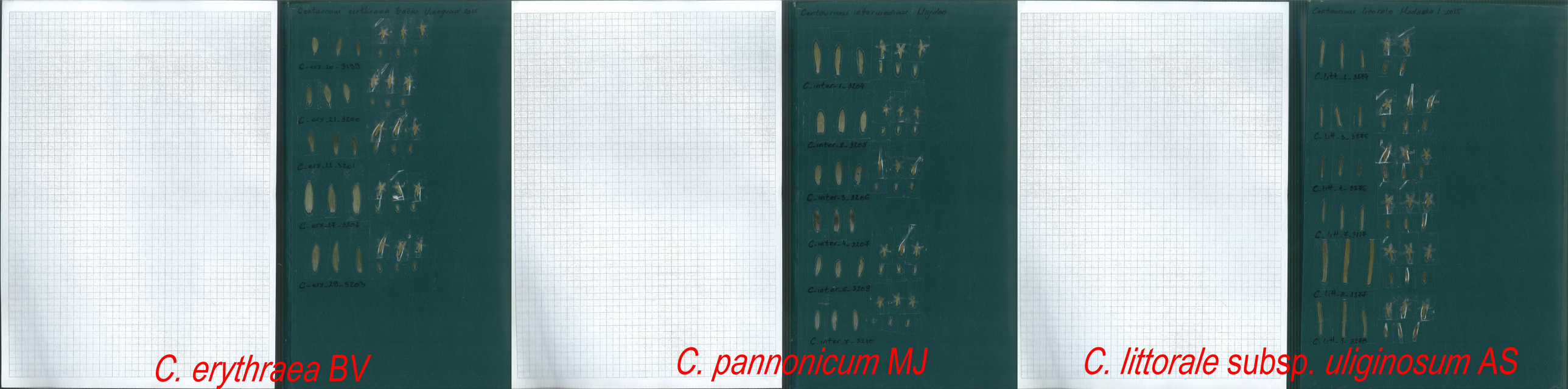


**Figure S5.** Representative photographs of analyzed leaves and flowers belonging to the three studied taxa.

**Table S1.** Confusion matrix depicting the performance of the LDA model in taxon discrimination. The model was constructed using the following morphometric features: length/width ratio of floral stalk leaves, calyx/corolla-tube ratio, length of corolla lobes, and cosine of the leaf tip angle. Performance was estimated based on the hold out set predictions in 10 times repeated 5-fold cross validation (entries are presented as percent average cell counts across resamples).

| **Reference**  **Prediction** | ***C. erythraea*** | ***C. littorale* subsp. *compressum*** | ***C. pannonicum*** |
| --- | --- | --- | --- |
| *C. erythraea* | 43.5 | 0.1 | 3.2 |
| *C. littorale* subsp. *compressum* | 0.7 | 36.8 | 1.6 |
| *C. pannonicum* | 0.2 | 2.7 | 11.3 |

**Table S2.** Raw morphometric data. (See Excel File)
